# Predictors of Poor Outcome Despite Successful Mechanical Thrombectomy of Anterior Circulation Large Vessel Occlusions Within 6 h of Symptom Onset

**DOI:** 10.3389/fneur.2020.00907

**Published:** 2020-09-04

**Authors:** Mahmoud H. Mohammaden, Christopher J. Stapleton, Denise Brunozzi, Ahmad E. Hussein, Eman M. Khedr, Gursant Atwal, Ali Alaraj

**Affiliations:** ^1^Department of Neurology, South Valley University Qena Faculty of Medicine, Qena, Egypt; ^2^Department of Neurology, Marcus Stroke and Neuroscience Center, Emory University School of Medicine, Grady Memorial Hospital, Atlanta, GA, United States; ^3^Department of Neurosurgery, College of Medicine, University of Illinois at Chicago, Chicago, IL, United States; ^4^Department of Neurology, Faculty of Medicine, Assiut University, Assiut, Egypt

**Keywords:** acute ischemic stroke, mechanical thrombectomy, internal carotid artery occlusion, distal clot migration, poor functional outcome

## Abstract

**Background and Purpose:** Successful reperfusion is a significant predictor of a good clinical outcome after mechanical thrombectomy (MT). However, some patients have a poor clinical outcome even with successful reperfusion. We aimed to study factors that predict a poor clinical outcome (90-day modified Rankin Scale ≥ 3) in patients with anterior circulation large vessel occlusion (LVO) treated by successful MT within 6 h of symptom onset.

**Methods:** We performed a retrospective review of a prospectively maintained MT database of all patients who underwent MT within 6 h of symptom onset for an anterior circulation LVO at our institution from May 2016 to June 2018. Uni- and multivariable analyses were performed to identify predictors of poor outcome.

**Results:** A total of 56 patients met the criteria for inclusion in this study. A poor outcome occurred in 31 (55.4%) patients. On univariate analysis, compared to patients with good clinical outcome, patients with poor outcome had higher mean baseline NIHSS scores (23.3 vs. 13.8, *P* < 0.001), were more likely to have internal carotid artery (ICA) occlusions (38.7 vs. 8%, *P* = 0.008), and had a higher incidence of distal clot migration (DCM) (48.4 vs. 8%, *P* = 0.028). Age, gender, other baseline clinical characteristics, MT technique, and incidence of hemorrhagic transformation did not differ between the two cohorts. On multivariable regression analysis, baseline NIHSS score [OR; 1.3, 95%CI [1.11–1.52], *P* = 0.001], site of occlusion (ICA) [OR; 8.9, 95%CI [1.3–60.9], *P* = 0.026], and DCM [OR; 5.77, 95%CI [1.09–30.69], *P* = 0.04] were independent predictors of poor outcome at 90-days.

**Conclusion:** Baseline NIHSS score, ICA occlusion, and DCM are independent predictors of a poor outcome after MT for anterior circulation LVO performed within 6 h of symptoms onset.

## Introduction

Stroke is the third leading cause of death in developed countries and the most common cause of permanent disability (1). Mechanical thrombectomy (MT) within 6 h of symptom onset is the standard treatment for acute ischemic stroke (AIS) due to anterior circulation emergent large vessel occlusion (ELVO) (2). The guidelines extended the eligibility of MT after the results of the Endovascular Therapy Following Imaging Evaluation for Ischemic Stroke (DEFUSE 3) (3) and the DWI or CTP Assessment with Clinical Mismatch in the Triage of Wake-Up and Late Presenting Strokes Undergoing Neurointervention with Trevo (DAWN) (4) trials up to 16 and 24 h, respectively in well-selected patients with clinical/imaging mismatch (2).

The outcome after MT depends mainly on the degree of recanalization, with the failure of recanalization is the strongest predictor of poor outcome (5). Despite the use of modern era devices and techniques for MT, which has enhanced the speediness and completeness of reperfusion of the downstream territory, the likelihood of a good functional outcome (modified Rankin Scale, mRS0-2) at 90 days was 46% with many procedures are futile (6).

In this study, we aimed to evaluate the predictors of poor functional outcome at 90-days in patients with successful recanalization within 6 h of symptom onset.

## Methods

### Patients

We performed a retrospective review of a prospectively maintained MT database at our tertiary comprehensive stroke center from May 2016 to June 2018. Patients were included in the study if they had anterior circulation LVO, presented within 6 h of symptom onset, and underwent MT with successful reperfusion defined as achievement of modified Thrombolysis In Cerebral Infarction (mTICI) of 2b-3. The institutional review board approved the study.

### Baseline Assessment

Patient demographics, stroke risk factors, baseline National Institute of Health Stroke Scale (NIHSS) score, time since last known well (LKW), and pre-procedure intravenous tissue plasminogen activator (IV-tPA) administration were reviewed and recorded as well as Alberta Stroke Program Early Computed Tomography Score (ASPECTS) which was assessed by a stroke neurologist across our database.

### Endovascular Procedure

The decision to perform MT was based on multidisciplinary discussions between the vascular neurology and neuroendovascular teams. All patients were treated with MT using stent-retrievers or contact aspiration techniques according to the most recent guidelines. Procedural data with regard to the site of occlusion; internal carotid artery (ICA) or middle cerebral artery (MCA), time from groin puncture to successful recanalization, and the numbers of passes were recorded. No proximal balloon guide catheter was used in any of those cases.

### Complication and Outcome

Hemorrhagic transformation was assessed by post-treatment head computed tomography (CT). Symptomatic intracranial hemorrhage (sICH) was defined as any ICH on follow-up CT leading to neurologic deterioration, as reflected by NIHSS score worsening of ≥ 4 points. Distal clot migration (DCM) to the same or new territory was identified from post-MT angiography and compared to pre-treatment CT angiography, with corresponding cerebral infarction on cross-sectional imaging. A 90-day mRS 3–6 was considered a poor outcome.

### Statistical Analysis

Quantitative variables were described as mean ± standard deviation (SD) after normality testing with the Shapiro-Wilk test. Categorical variables were presented as frequencies and percentages. For the univariate analysis, data were compared using Student's *t*-tests for continuous variables and chi-square or Fisher's exact tests for categorical variables as appropriate. On multivariable regression analysis, all variables with *P* < 0.1 in the univariate analyses were included in the regression model to determine the independent predictors of poor outcome. Statistical significance was set at *P* < 0.05. Statistical evaluations were performed using STATA/SE software, version 10.1 (StataCorp, College Station, TX).

## Results

### Baseline Characteristics

A total of 56 patients met the criteria for inclusion in this study. Poor outcomes occurred in 31 (55.4%) patients, of whom 17 (54.8%) were females, with a mean age of 68.4 years. There were no significant differences in baseline medical comorbidities between patients with good vs. poor 90-day functional outcomes. LKW to puncture time, ASPECTS, and pre-procedure IV-tPA also did not differ between the two groups. The mean ± SD baseline NIHSS score was significantly higher in patients with poor 90-day outcomes (21.3 ± 5.3 vs. 13.8 ± 5.7, *P* < 0.001) (Table 1).

**Table 1:** Baseline, clinical characteristics, angiographic findings between good (90-day mRS 0–2) and poor outcome (90-day mRS 3–6).

	**Good outcome**	**Poor outcome**	***p*-value**
	***n* = 25**	***n* = 31**	
**Demographics and risk factors n (%)**			
Age years mean ± SD	65.2 ± 15.1	68.4 ± 14	0.40
Female	10 (40)	17 (54.8)	0.27
Atrial fibrillation	12 (48)	12 (38.7)	0.49
Hypertension	20 (78.8)	23 (80)	0.61
Diabetes mellitus	4 (16)	7 (22.6)	0.54
**Clinical and procedural characteristics n (%)**			
LKW-puncture (min) mean ± SD	215.8 ± 75	228.2 ± 77.9	0.55
Baseline NIHSS score mean ± SD	13.8 ± 5.7	21.3 ± 5.3	**<0.001**
ASPECTS mean ± SD	9.4 ± 0.7	9 ± 1.1	0.14
Pre-procedure IV-tPA	17 (68)	23 (74.2)	0.61
Site of occlusion:			
Internal carotid artery	2 (8)	12 (38.7)	
Middle cerebral artery	23 (92)	19 (61.3)	**0.008**
Tandem occlusions	1 (4)	6 (19.4)	0.09
MT technique			
Stent-retriever	17 (68)	25 (80.6)	
Contact Aspiration	8 (32)	6 (19.4)	0.28
Number of passes mean ± SD	1.9 ± 1.4	2.3 ± 1.8	0.61
Hemorrhagic transformation	7 (28)	14 (45.2)	0.19
sICH	2 (8)	6 (19.4)	0.23
Distal clot migration	5 (20)	15 (48.4)	**0.028**
NIHSS score at 24 h mean ± SD	3.2 ± 3.7	17.7 ± 5.5	**<0.001**

*LKW, last known well; NIHSS score, national institute of health stroke scale score; ASPECTS, Alberta Stroke Program Early Computed Tomography score; IV-tPA, intravenous tissue plasminogen activator; MT, mechanical thrombectomy; sICH, symptomatic intracranial hemorrhage. Boldface indicates statistical significance*.

### Procedural Findings

Patients with poor outcomes were more likely to have ICA occlusions (38.7 vs. 8%, *P* = 0.008). There was a trend of higher rates of tandem occlusions in patients with poor outcome (19.4 vs. 4%, *P* = 0.09). The mean time from puncture to successful reperfusion (mTICI 2b/3) was comparable between the two groups (51.1 ± 36.7 vs. 37.1 ± 23.3, *P* = 0.16) (Table 1).

### Complications and Outcome

There were no significant differences between the two groups in the overall rates of hemorrhagic transformation (45.2 vs. 28%, *P* = 0.19) or sICH (19.4 vs. 8%, *P* = 0.23). Patients with poor outcomes had higher rates of DCM (48.4 vs. 20%, *P* = 0.028) and higher mean NIHSS scores at 24 h post-procedure (17.7 ± 5.5 vs. 3.2 ± 3.7, *P* < 0.001) (Table 1).

### Predictors of Poor Outcome

On multivariable regression analysis, baseline NIHSS score [OR; 1.3, 95%CI [1.11–1.52], *P* = 0.001], site of occlusion (ICA) [OR; 8.9, 95% CI [1.3–60.9], *P* = 0.026], and DCM [OR; 5.77, 95% CI [1.09–30.69], *P* = 0.04] were independent predictors of poor outcome (Table 2).

**Table 2:** Multivariate analysis for predictors of poor outcome.

	**Odds ratio**	**95% confidence interval**	***p*-value**
Baseline NIHSS score	1.3	1.11–1.52	**0.001**
Site of occlusion (ICA)	8.9	1.3–60.9	**0.026**
Distal clot migration (DCM)	5.77	1.09–30.69	**0.04**

*Boldface indicates statistical significance*.

## Discussion

Our study demonstrated that baseline NIHSS score, ICA occlusion, and DCM are independent predictors of poor functional outcome despite successful recanalization after MT within 6 h of symptom onset. Our finding of higher NIHSS score and its association with the poor functional outcome is consistent with several previous studies which evaluated the prognostic factors of outcome after MT (7–9). A high NIHSS on admission was also associated with both proximal ICA occlusion and poor collateral circulation (10).

**Figure 1:**
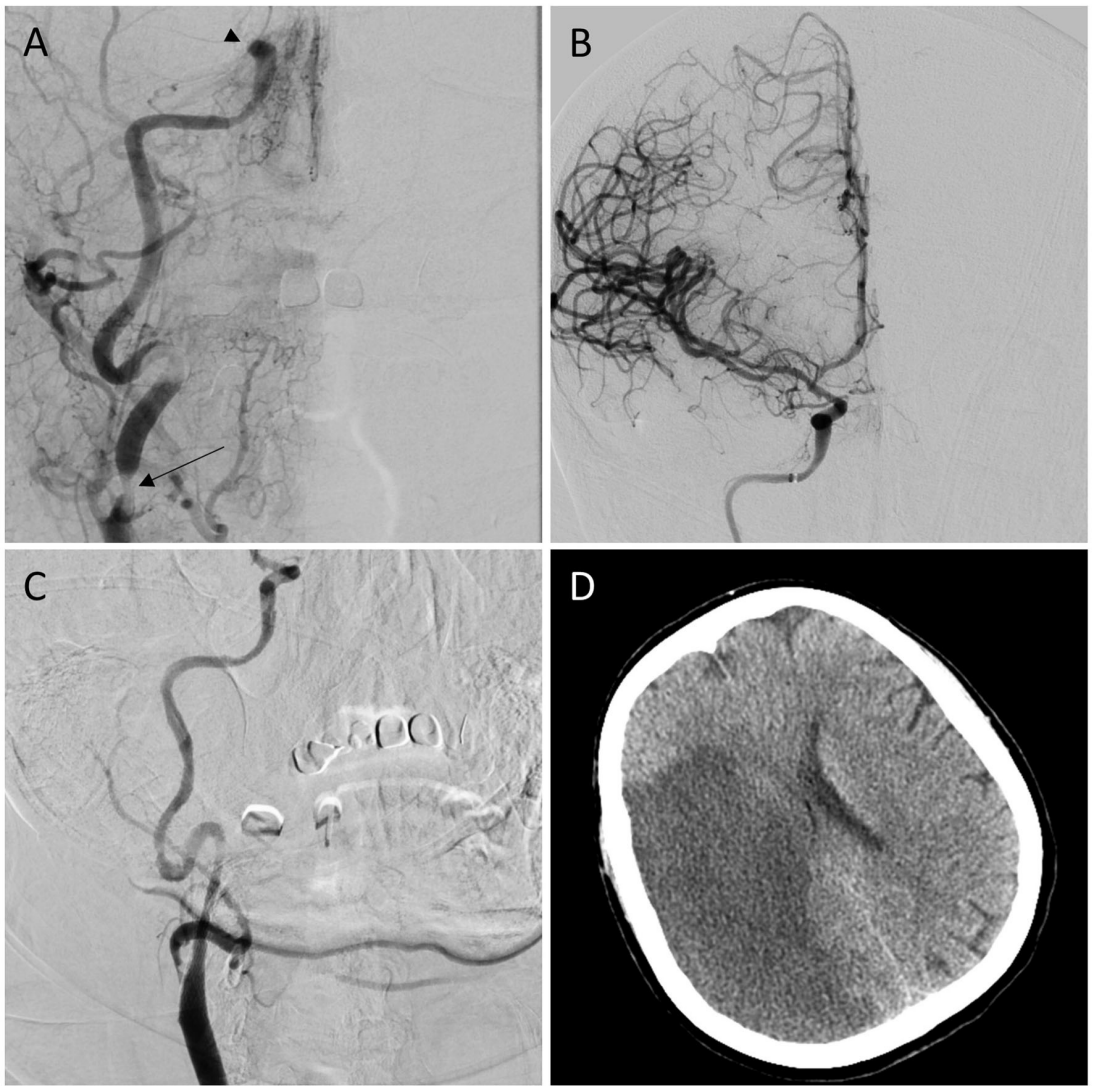
**(A)** Anterior-posterior (A–P) projection of cerebral angiogram shows the right internal carotid artery terminus occlusion (arrowhead) with stenosis in the cervical portion (arrow). **(B)** A–P projection of cerebral angiogram after mechanical thrombectomy shows Complete recanalization (mTICI 3). **(C)** A–P projection after carotid artery stenting shows successful angioplasty and stenting of cervical carotid artery stenosis. **(D)** Corresponding CT brain shows cerebral infarction at the right middle cerebral artery territory.

Previous studies have shown that IV-tPA was associated with better recanalization of MCA occlusions as compared to more proximal ICA occlusions, (11) an observation that has held true even in the era of endovascular intervention, which can be explained by the fact that ICA occlusions are more complex than MCA occlusions as they can be either a single artery occlusion or a tandem lesion. Moreover, ICA terminus occlusion often extends into both the M1 segment of MCA and/or A1 segment of the anterior cerebral artery and usually exhibit large clot burdens and impair collateral circulations (12). Tandem occlusions involve both the cervical ICA and either the intracranial ICA (Figure 1), MCA-M1, or ACA-A1. Our results did not report a significant difference in the outcome of MT in patients with tandem occlusion and those without which indicates that ICA occlusion *per se* is a predictor of poor outcome.

In this study, we found that DCM either to the same or to an unaffected territory resulted in a poor functional outcome. We believe this is due to the compromise of collateral vessels that result in loss of salvageable brain tissue (13). Recanalization via stent-retrievers or aspiration thrombectomy did not result in different outcomes nor did time to recanalization or the number of thrombectomy passes (14, 15).

Finally, our study demonstrated that pre-MT IV-tPA administration did not improve outcomes after MT, which is consistent with a recent randomized controlled trial that reported non-inferiority of direct MT without prior IV-tPA to MT preceded by IV-tPA in terms of functional outcome (16).

Our study has all the limitations inherent to any retrospective analysis. Moreover, the small sample size might limit the generalizability of our results. Also, there was no core laboratory adjudication of final reperfusion grades. The strength of our study is the inclusion of only patients who achieved successful reperfusion.

## Conclusions

Our study demonstrates that baseline NIHSS score, ICA occlusion, and DCM are independent predictors of poor functional outcome in patients undergoing MT within 6 h of symptom onset. Identifying factors which lead to poor clinical outcome and modification of these factors if possible can result in an improvement of functional independence of patients with AIS treated with MT. Large multicenter prospective studies are warranted.

## Data Availability Statement

The raw data supporting the conclusions of this article will be made available by the authors, without undue reservation.

## Ethics Statement

The studies involving human participants were reviewed and approved by the institutional review board of the University of Illinois at Chicago Hospital. Written informed consent for participation was not required for this study in accordance with the national legislation and the institutional requirements.

## Author Contributions

MM: study conception, design of the work, data acquisition, interpretation of data, and drafting of the manuscript. CS, DB, AH, EK, and GA: critical revision of the manuscript. AA: interpretation of data and critical revision of the manuscript. All authors contributed to the article and approved the submitted version.

## Conflict of Interest

AA: Consultant: Cerenovus. The remaining authors declare that the research was conducted in the absence of any commercial or financial relationships that could be construed as a potential conflict of interest.
